# Off-pump coronary artery bypass grafting with clampless aortic anastomosis devices: Aortic sealing devices versus automated anastomosis punching

**DOI:** 10.1016/j.xjtc.2024.01.010

**Published:** 2024-01-23

**Authors:** Mustafa Gerçek, Tomislav Skuljevic, Marcus-André Deutsch, Jan Gummert, Jochen Börgermann

**Affiliations:** a Heart Center Duisburg, Clinic for Cardiac Surgery and Pediatric Cardiac Surgery, Duisburg, Germany; b Ruhr-University Bochum, Bochum, Germany; c Herz- und Diabeteszentrum NRW, Clinic for Thoracic and Cardiovascular Surgery, Bad Oeynhausen, Germany

**Keywords:** clampless cardiac surgery, OPCAB, clampless aortic anastomosis device, stroke

## Abstract

**Objectives:**

Clampless aortic anastomosis devices aim to lower stroke risk in off-pump coronary artery bypass grafting. Two main strategies for clampless anastomosis devices emerged with automated anastomosis punching and aortic sealing devices, prompting the question of perioperative outcome differences.

**Methods:**

All consecutive patients undergoing elective off-pump coronary artery bypass grafting with a clampless aortic anastomosis device between September 2014 and December 2021 in 2 centers were retrospectively included. Cohorts were divided by the use of an automated anastomosis punching device or an aortic sealing device to achieve proximal anastomosis on the ascending aorta. To reach group comparability propensity score matching was performed. The primary end point was defined as a composite of all-cause mortality, stroke and rethoracotomy. Secondary end points were perioperative outcome parameters.

**Results:**

A total of 3703 patients were enrolled of whom 575 and 3128 were included in the automated anastomosis punching and the aortic sealing device group, respectively. By propensity score matching a total of 1150 patients were included with 575 in each group. The primary composite endpoint showed no significant difference with 6.3% versus 5.9% events (odds ratio, 0.9; 95% confidence interval, 0.58-1.53, *P* = .81). All-cause mortality (*P* = .36), stroke (*P* = .81), and rethoracotomy (*P* = .89) also exhibit no disparity. Operation time was significantly longer in the aortic sealing device cohort with 220.0 ± 50.8 minutes and 204.6 ± 53.8 minutes (*P* < .01).

**Conclusions:**

Clampless aortic anastomosis strategies aortic sealing device and automated anastomosis punching did not differ in perioperative outcome parameters, whereas the implementation of aortic sealing devices were associated with a prolonged operation time without inducing any inferior clinical outcome.

## Graphical Abstract

**Figure:**
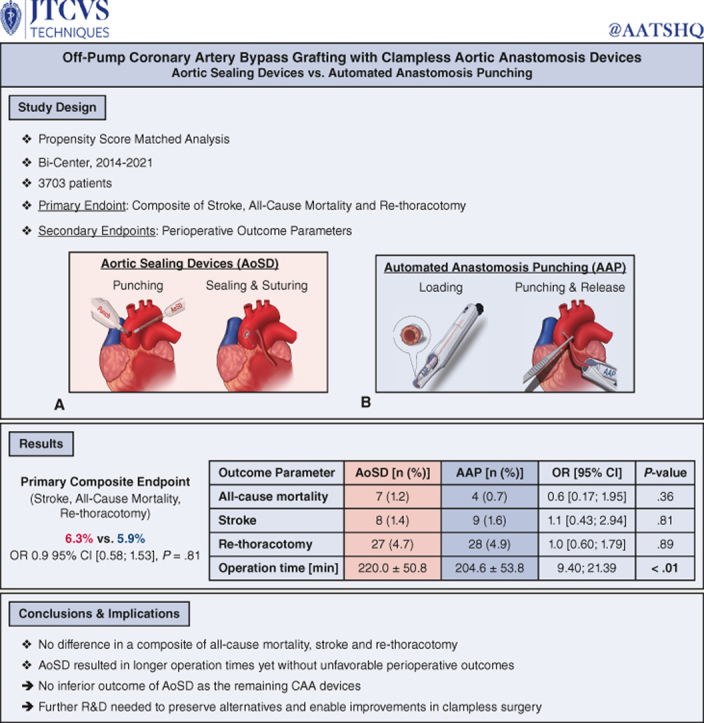

**Figure:**
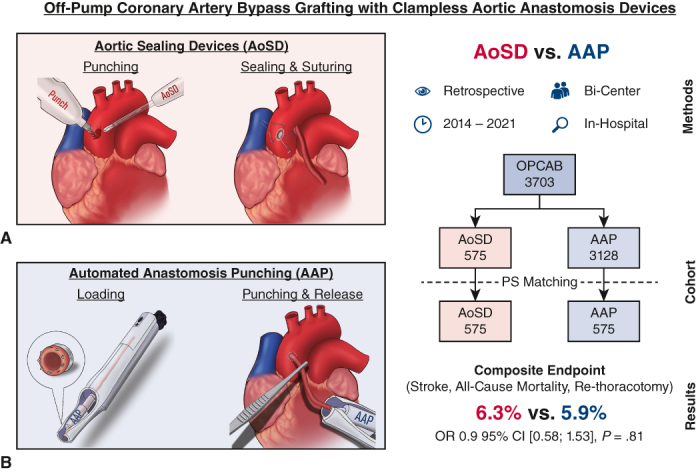
Clampless bypass surgery with aortic sealing devices versus automated anastomosis punching.

**Central Message**

The CAA strategies AoSD and AAP do not differ in perioperative outcome, whereas the implementation of AoSD is associated with a prolonged operation time without inducing any inferior clinical outcome.

**Perspective**

Despite the limitation of clampless anastomosis to one strategy only with AoSD, the in-hospital outcome regarding a composite of all-cause mortality, stroke, and rethoracotomy and perioperative outcomes was not inferior to the former second major strategy, AAP. Nevertheless, further research improving current and establishing new strategies is mandatory to shape the future of clampless bypass surgery.

Coronary artery bypass grafting (CABG) represents the foremost and first-line therapy for the surgical treatment of multivessel coronary artery disease, given its repeatedly proven safety and superiority in long-term survival outcomes.^1^, ^2^, ^3^ In addition to conventional CABG surgery involving extracorporeal circulation (ECC), cardiac arrest, crossclamping, and partial aortic clamping, off-pump coronary artery bypass grafting (OPCAB) is a well-established surgical strategy achieving myocardial revascularization while reducing stroke risk by avoiding ECC and in particular by minimizing aortic manipulation.^4^^,^^5^ Since cerebral injuries remain a major complication in bypass surgery^6^ and are heavily associated with aortic manipulation and calcification,^7^, ^8^, ^9^ the no-aortic touch technique remains the best choice by, for example, using total arterial revascularization.^6^^,^^10^, ^11^, ^12^, ^13^ However, due to the presence of patient-dependent reasons, venous grafting on the ascending aorta is still and will most likely remain the primary strategy in bypass surgery.

Thus, in the ambition to discover methods to minimize aortic manipulation in these cases, clampless aortic anastomosis (CAA) devices were developed. In the history of CAA techniques, 2 main approaches have surfaced: aortic sealing devices (AoSD)^4^^,^^14^^,^^15^ and automated anastomosis punching (AAP).^16^^,^^17^ AoSDs use a sealing system to control aortic bleeding and to facilitate manual anastomosis suturing, whereas AAP devices use a system that automatically punches and releases the anastomosis into the ascending aorta, eliminating the need for sutured anastomoses on the ascending aorta (Figure 1). Although current guidelines provide detailed recommendations on conduit selection, vessel harvesting, or minimally invasive techniques, the only general instruction regarding the ascending aorta is to minimize any kind of aortic manipulation.^1^ In particular, the guidelines recommend avoiding multiple manipulations, to reduce the risk of stroke.^5^^,^^18^^,^^19^ Therefore, single or no clamping of the aorta might yield a better outcome.^4^^,^^20^ A further statement on any CAA system is still missing.

**Figure 1:**
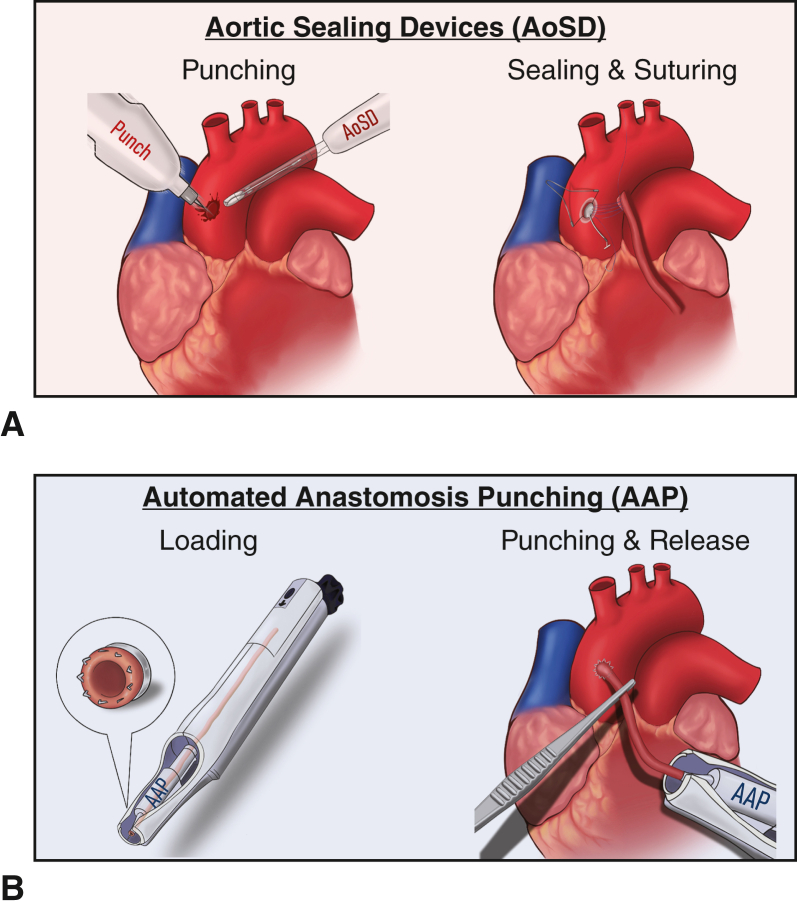
Clampless aortic anastomosis devices. A, Aortic sealing devices; (B) automated anastomosis punching devices. *AoSD*, Aortic sealing device; *AAP*, automated anastomosis punching.

Although both systems, AoSD and AAP, have demonstrated their safety and effectiveness,^14^^,^^17^^,^^21^ data comparing the perioperative outcomes of the 2 systems remain limited. Since AAP devices were withdrawn from the market 2021 and are no longer available,^22^ focusing the field of OPCAB surgery to a single strategy, this situation offered an opportunity to compare device-related outcomes in the same setting, enabling us to gain a better understanding of the CAAs and to draw the further path of clampless bypass surgery.

## Patients and Methods

### Patient Recruitment

All consecutive patients who underwent isolated OPCAB between 2018 (onset of OPCAB program) and 2021 in the Herzzentrum Duisburg (Duisburg, Germany) (Center 1) and between 2014 and 2021 in the Herz- und Diabeteszentrum NRW (Bad Oeynhausen, Germany) (Center 2) were retrospectively recruited. Exclusion criteria were defined as patients who underwent urgent or emergency surgery, patients without the conduction of a central anastomosis on the ascending aorta, any concomitant surgical procedure, and patients with missing data. Patients with concomitant amputation of the left atrial appendage were not excluded but adjusted during matching.

The institutional review board or equivalent ethics committee of the Ärztekammer Nordrhein (No. 90/2023; date: December 4. 2023) and the Ruhr University Bochum (No. 2023-1092; date: November 7, 2023) approved the study protocol and publication of data.

### Outcome and Subgroups

The primary outcome was defined as a composite of all-cause mortality, stroke, and rethoracotomy. The term “rethoracotomy” is used to denote the necessity for surgical reintervention in the perioperative period due to, for example, major bleeding or cardiac tamponade. Secondary end points were defined as the isolated parameters of the composite end point. Additional secondary end points are perioperative outcome parameters and cardiac enzymes. The digital archives of the data-management systems of the participating centers posed as the source of information. To assess subgroup differences, subgroup analysis of the participating centers, analysis regarding cardiac function, and analysis regarding age-based disparities were performed.

### Surgical Technique

CABG was performed via the off-pump technique. Surgical access was achieved through median sternotomy, whereas well-established grafts such as the left and/or right internal mammary artery, radial artery, and saphenous vein were employed. After the pericardial exposure, the surgeon carefully assessed the ascending aorta to ensure the use of CAA devices. For the AoSD group, the Heartstring III System (Getinge Group) was chosen, whereas for the AAP group, the PAS-Port System (B. Braun) was used. The AoSD or AAP device was then determined by the surgeon’s preference or availability. Partial aortic clamping acted as a bail-out strategy if any complication occurred. Except for the choice of the CAA device, surgery was conducted in the same manner. Transit time flow measurement was used for quality control of the anastomoses. Suction systems with autotransfusion of the lost blood were used per standard protocol in both cohorts. Postoperatively, all patients received a dual-antiplatelet therapy consisting of acetylsalicylic acid 100 mg and clopidogrel 75 mg. Patients suffering from perioperative atrial fibrillation were treated with acetylsalicylic acid 100 mg and oral anticoagulation. The dual-antiplatelet therapy was recommended for a duration of 6 months. Afterwards, a transition to monoantiplatelet therapy with acetylsalicylic acid 100 mg was advised.

### Statistical Analysis

Statistical analysis was performed via the SPSS Software (Version 28; IBM Corp) and R (Version 4.2.2; R Core Team). Categorical variables are given as absolute and relative frequencies and continuous variables as means with standard deviations. Categorical variables were compared with the use of the χ^2^ test or Fisher exact test, and continuous variables were compared with the use of unpaired *t* tests or the Mann–Whitney *U* test in the unmatched cohorts, and with paired *t* tests in the propensity score–matched cohorts. A logistic regression model was applied to primary composite end point and the isolated parameters of the composite end point. Parameter estimates are given with their odds ratios (ORs), 95% confidence intervals (95% CI), and the corresponding *P* value.

Since the CAA device (AoSD or AAP) was chosen in a nonrandomized fashion, we used 1:1 propensity score (PS) matching by 40 baseline or operative characteristics (Table 1) by nearest neighbor matching with a caliper of 0.2. To conclude, balance of baseline covariates was assessed by computing the standardized mean difference (balance achieved, if < |0.1|).

**Table 1:** Baseline and operative characteristics used for PS matching in the unmatched and PS-matched cohorts

Variable	Unmatched cohort				PS–matched cohort			
	AoSD, n (%) 575 (100.0)	AAP, n (%) 3128 (100.0)	OR (95% CI)	SMD	AoSD, n (%) 575 (100.0)	AAP, n (%) 575 (100.0)	OR (95% CI)	SMD
Center			9.0 (7.32-11.17)	−0.71			1.1 (0.90-1.44)	−0.06
Duisburg	246 (42.8)	239 (7.6)			246 (42.8)	228 (39.7)		
Bad Oeynhausen	329 (57.2)	2889 (92.4)			329 (57.2)	347 (60.4)		
Patient characteristics								
Gender (female)	114 (19.8)	615 (19.7)	1.0 (0.79-1.24)	<0.01	114 (19.8)	113 (19.7)	1.0 (0.74-1.32)	<0.01
Age, y	69.3 ± 9.0	69.4 ± 9.1	−0.97 to 0.64	−0.02	69.3 ± 9.0	69.2 ± 9.4	−0.99 to 1.14	<0.01
Height, cm	172.8 ± 9	172.2 ± 8.8	−0.18 to 1.40	0.07	172.8 ± 9.0	172.9 ± 8.8	−1.08 to 0.98	<–0.01
Weight, kg	86.7 ± 16.3	85.0 ± 16.1	0.22-3.10	0.10	86.7 ± 16.3	86.9 ± 16.8	−2.12 to 1.70	−0.01
BMI, kg/m^2^ BSA	29.0 ± 4.8	28.6 ± 4.7	−0.07 to 0.77	0.07	29.0 ± 4.8	29.0 ± 4.9	−0.62 to 0.50	−0.01
NYHA (class)	2.4 ± 0.8	2.1 ± 0.8	0.19-0.34	0.36	2.4 ± 0.8	2.4 ± 0.8	−0.07 to 0.10	0.03
CCS (class)	1.7 ± 1.1	1.5 ± 1.1	0.12-0.33	0.20	1.7 ± 1.1	1.7 ± 1.2	−0.05 to 0.22	0.08
Preoperative rhythm (AF)	28 (4.9)	88 (2.8)	0.6 (0.37-0.87)	0.10	28 (4.9)	29 (5.0)	1.0 (0.61-1.77)	<–0.01
DM	243 (42.3)	1247 (39.9)	0.9 (0.76-1.08)	0.05	243 (42.3)	251 (43.7)	1.1 (0.84-1.34)	−0.03
HLP	512 (89.0)	2927 (93.6)	1.8 (1.33-2.41)	−0.15	512 (89.0)	524 (91.1)	1.3 (0.86-1.86)	−0.07
AHT	517 (89.9)	2837 (90.7)	1.1 (0.81-1.47)	−0.03	517 (89.9)	516 (89.7)	1.0 (0.67-1.44)	<0.01
COPD	77 (13.4)	329 (10.5)	0.8 (0.58-0.99)	0.08	77 (13.4)	70 (12.2)	0.9 (0.63-1.27)	0.04
pAD	71 (12.4)	397 (12.7)	1.0 (0.79-1.35)	−0.01	71 (12.4)	80 (13.9)	1.2 (0.81-1.62)	−0.05
cAD	68 (11.8)	343 (11.0)	0.9 (0.70-1.21)	0.03	68 (11.8)	59 (10.3)	0.9 (0.59-1.23)	0.05
Medication								
ASA	474 (82.4)	2585 (82.6)	1.0 (0.80-1.28)	<–0.01	474 (82.4)	475 (82.6)	1.0 (0.75-1.37)	<–0.01
P2Y12 inhibitor	58 (10.1)	457 (14.6)	1.5 (1.14-2.04)	−0.15	58 (10.1)	58 (10.1)	1.0 (0.68-1.47)	0
Statins	458 (79.7)	2306 (73.7)	0.7 (0.58-0.89)	0.15	458 (79.7)	470 (81.7)	1.1 (0.85-1.53)	−0.05
ACE	233 (40.5)	1548 (49.5)	1.4 (1.20-1.72)	−0.18	233 (40.5)	234 (40.7)	1.0 (0.80-1.27)	<−0.01
ARB	234 (40.7)	856 (27.4)	0.6 (0.46-0.66)	0.27	234 (40.7)	240 (41.7)	1.0 (0.83-1.32)	−0.02
Beta blockers	339 (59.0)	2087 (66.7)	1.4 (1.16-1.67)	−0.16	339 (59.0)	326 (56.7)	0.9 (0.72-1.15)	0.05
Diuretics	198 (34.4)	1159 (37.1)	1.1 (0.93-1.35)	−0.06	198 (34.4)	185 (32.2)	0.9 (0.71-1.15)	0.05
Echocardiography								
Preoperative EF (%)	53.0 ± 8.4	55.0 ± 10.0	−2.92 to −1.18	−0.24	53.0 ± 8.4	52.7 ± 9.8	−0.74 to 1.37	0.04
Preoperative AR (grade)	0.1 ± 0.4	0.2 ± 0.4	−0.07 to 0.01	−0.07	0.1 ± 0.4	0.1 ± 0.4	−0.04 to 0.05	0.03
Preoperative AS (grade)	0.1 ± 0.3	0.0 ± 0.3	−0.01 to 0.04	0.07	0.1 ± 0.3	0.1 ± 0.3	−0.04 to 0.04	0.01
Preoperative MR (grade)	0.4 ± 0.6	0.4 ± 0.6	−0.07 to 0.04	−0.02	0.4 ± 0.6	0.3 ± 0.6	−0.05 to 0.10	0.05
Preoperative MS (grade)	0.0 ± 0.1	0 ± 0.1	−0.01 to 0.01	0.05	0.0 ± 0.1	0.01 ± 0.1	−0.02 to 0.01	−0.02
Preoperative TR (grade)	0.2 ± 0.5	0.2 ± 0.5	−0.08 to 0.00	−0.08	0.2 ± 0.5	0.2 ± 0.4	−0.05 to 0.05	0.01
Preoperative TS (grade)	0.0 ± 0.0	0 ± 0.01	−0.01 to 0.00	−0.02	0.0 ± 0.0	0.0 ± 0.0		0
Operative characteristics								
BIMA	90 (15.7)	451 (14.4)	0.9 (0.71-1.16)	0.03	90 (15.7)	82 (14.3)	0.9 (0.65-1.24)	0.04
Radialis	3 (0.5)	3 (0.1)	0.2 (0.04-0.91)	0.06	3 (0.5)	3 (0.5)	1.0 (0.20-4.98)	0
TAR	1 (0.2)	3 (0.1)	0.6 (0.06-5.31)	0.02	1 (0.2)	1 (0.2)	1.0 (0.06-16.03)	0
LAA amp.	110 (19.1)	902 (28.8)	1.7 (1.37-2.14)	−0.25	110 (19.1)	109 (19.0)	1.0 (0.74-1.33)	<0.01
Ablation	17 (3.0)	21 (0.7)	0.2 (0.12-0.42)	0.13	17 (3.0)	18 (3.1)	1.1 (0.54-2.08)	−0.01
Distal anastomosis (no)	3.0 ± 0.8	3.0 ± 0.8	−0.13 to 0.00	−0.08	3.0 ± 0.8	3.0 ± 0.8	−0.11 to 0.07	−0.02
Arterial anastomosis (no)	1.2 ± 0.6	1.2 ± 0.6	−0.04 to 0.07	0.04	1.2 ± 0.6	1.2 ± 0.6	−0.05 to 0.09	0.03
Venous anastomosis (no)	1.7 ± 0.7	1.8 ± 0.7	−0.15 to −0.02	−0.12	1.7 ± 0.7	1.8 ± 0.7	−0.12 to 0.04	−0.05
Grafts (no)	2.2 ± 0.4	2.2 ± 0.5	−0.04 to 0.04	<0.01	2.2 ± 0.4	2.1 ± 0.4	−0.04 to 0.06	0.05
Arterial grafts (no)	1.1 ± 0.4	1.1 ± 0.4	−0.02 to 0.06	0.06	1.1 ± 0.4	1.1 ± 0.4	−0.04 to 0.06	0.03
Venous grafts (no)	1.0 ± 0.2	1.1 ± 0.3	−0.05 to −0.01	−0.12	1.0 ± 0.2	1.0 ± 0.2	−0.02 to 0.02	0.03

*PS*, Propensity score; *AoSD*, aortic sealing device; *AAP*, automated anastomosis punching; *OR*, odds ratio; *95% CI*, 95% confidence interval; *SMD*, standardized mean difference; *BMI*, body mass index; *NYHA*, New York Heart Association; *CCS*, Canadian Cardiovascular Society; *AF*, atrial fibrillation; *DM*, diabetes mellitus; *HLP*, hyperlipidemia; *AHT*, arterial hypertension; *COPD*, chronic obstructive pulmonary disease; *pAD*, peripheral artery disease; *cAD*, cerebral artery disease; *ASA*, acetylsalicylic acid; *ACE*, angiotensin-converting enzyme inhibitor; *ARB*, angiotensin II receptor blocker; *EF*, ejection fraction; *AR*, aortic regurgitation; *AS*, aortic stenosis; *MR*, mitral regurgitation; *MS*, mitral stenosis; *TR*, tricuspid regurgitation; *TS*, tricuspid stenosis; *BIMA*, bilateral internal mammary artery; *TAR*, total arterial revascularization; *LAA amp*., left atrial appendage amputation.

## Results

### Cohort

**Figure 2:**
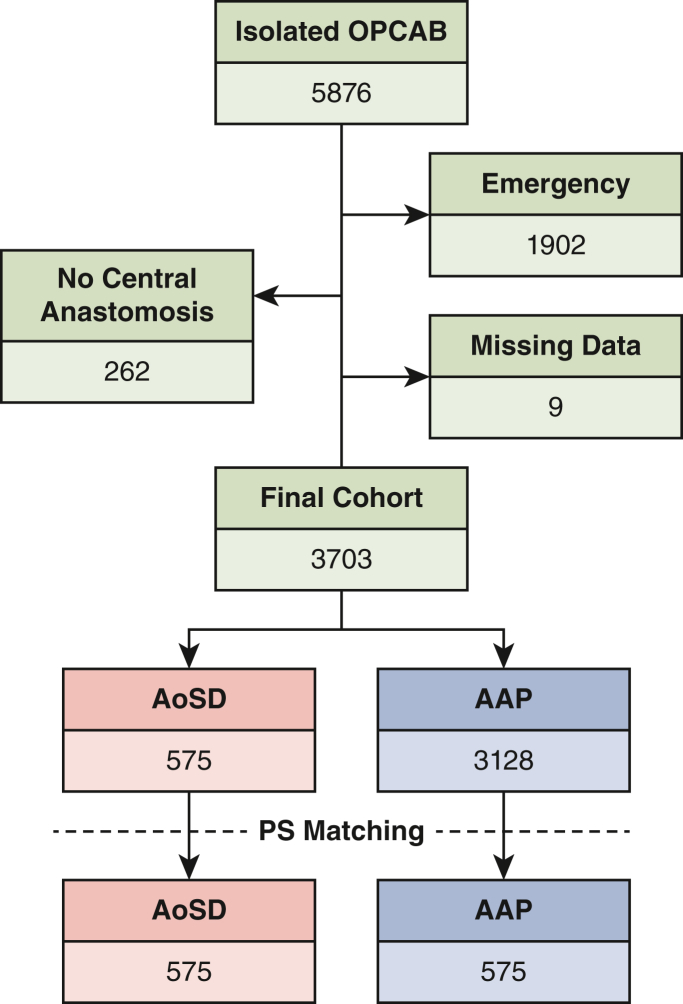
Patient-selection process. *OPCAB*, Off-pump coronary artery bypass grafting; *AoSD*, aortic sealing devices; *AAP*, automated anastomosis punching; *PS*, propensity score.

A total of 5876 patients who underwent isolated CABG could be identified. As defined by the exclusion criteria, 1902 not electively operated patients, 262 patients without central anastomosis on the ascending aorta, and 9 patients with missing data were excluded from the study (Figure 2). Thus, 3703 patients were enrolled, of whom 3128 and 575 were included in the AAP and the AoSD group, respectively. By PS matching, a total of 1150 patients were included with 575 patients in each group. Baseline characteristics used for PS matching consisted of information regarding general patient-based variables, medication, echocardiography, and operative parameters. In the unmatched cohorts, 16 variables presented a standardized mean difference of > |0.1|, whereas a standardized mean difference of < |0.1| in all parameters could be achieved by PS matching. The parameters in the unmatched and matched cohorts are illustrated in Table 1.

### Primary End Point

The composite end point consisting of all-cause mortality, stroke, and rethoracotomy showed no significant difference in the perioperative period between the AoSD and AAP cohort with 6.3% versus 5.9% events (OR, 0.9; 95% CI, 0.58-1.53, *P* = .81), respectively (Table 2, logistic regression in the unmatched cohort in Table E1).

**Table 2:** Perioperative outcome of the AoSD and AAP groups in the unmatched and matched cohorts

Variable	Unmatched cohort				Propensity score–matched cohort			
	AoSD, n (%) 575 (100.0)	AAP, n (%) 3128 (100.0)	OR (95% CI)	*P* value	AoSD, n (%) 575 (100.0)	AAP, n (%) 575 (100.0)	OR (95% CI)	*P* value
Composite end point	36 (6.3)	143 (4.6)	0.7 (0.49-1.05)	.08	36 (6.3)	34 (5.9)	0.9 (0.58-1.53)	.81
All-cause mortality	7 (1.2)	18 (0.6)	0.5 (0.20-1.13)	.08	7 (1.2)	4 (0.7)	0.6 (0.17-1.95)	.36
Stroke	8 (1.4)	32 (1.0)	0.7 (0.34-1.60)	.43	8 (1.4)	9 (1.6)	1.1 (0.43-2.94)	.81
Rethoracotomy	27 (4.7)	116 (3.7)	0.8 (0.51-1.20)	.26	27 (4.7)	28 (4.9)	1.0 (0.60-1.79)	.89
Operation time, min	220.0 ± 50.8	200.2 ± 47.5	15.47-24.01	**<.01**	220.0 ± 50.8	204.6 ± 53.8	9.40-21.39	**<.01**
Dialysis	17 (3.0)	123 (3.9)	1.3 (0.80-2.24)	.26	17 (3.0)	21 (3.7)	1.2 (0.65-2.38)	.51
Need for ICA	21 (3.7)	95 (3.0)	0.8 (0.51-1.34)	.44	21 (3.7)	20 (3.5)	1.0 (0.51-1.77)	.87
CPR	15 (2.6)	54 (1.7)	0.7 (0.37-1.17)	.15	15 (2.6)	12 (2.1)	0.8 (0.37-1.72)	.56
IABP	3 (0.5)	13 (0.4)	0.8 (0.23-2.80)	.72	3 (0.5)	5 (0.9)	1.7 (0.40-7.03)	.48
ECLS	5 (0.9)	14 (0.5)	0.5 (0.18-1.43)	.19	5 (0.9)	2 (0.4)	0.4 (0.08-2.06)	.26
PE	18 (3.1)	63 (2.0)	0.6 (0.37-1.08)	.09	18 (3.1)	19 (3.3)	1.1 (0.55-2.04)	.86
ICU stay, d	2.4 ± 5.7	2.4 ± 5.1	−0.45 to 0.46	.97	2.4 ± 5.7	2.6 ± 5.5	−0.89 to 0.43	.50
Enzymes								
CK_max_, U/L	771.5 ± 928.4	826.8 ± 1018.4	−144.93 to 34.24	.23	771.2 ± 929.4	849.6 ± 934.3	−185.93 to 29.21	.15
CK-MB_max_, U/L	32.5 ± 51.7	27.4 ± 61.2	−0.59 to 10.73	.08	32.5 ± 53.2	33.5 ± 59.7	−5.62 to 9.61	.61
Troponin_max_, pg/mL	3379.3 ± 15,011.8	5639.8 ± 24,290.1	−4315.13 to −205.69	**.03**	3379.3 ± 15,011.8	3866.4 ± 17,014.9	−2335.29 to 1361.15	.61
Creatinine_max_, mg/dL	1.2 ± 1.0	1.4 ± 1.1	−0.34 to −0.16	**<.01**	1.2 ± 1.0	1.2 ± 0.8	−0.15 to 0.06	.38

*P* values < .05 presented in bold.

Composite end point = all-cause mortality, stroke, rethoracotomy. *AoSD*, Aortic sealing device; *AAP*, automated anastomosis punching; *OR*, odds ratio; *95% CI*, 95% confidence interval; *ICA*, invasive coronary angiography; *CPR*, cardiopulmonary resuscitation; *IABP*, intra-aortic balloon pump; *ECLS*, extracorporeal life support; *PE*, pericardial effusion; *ICU*, intensive care unit; *CK*, creatinine kinase; *CK-MB*, creatinine kinase isoenzyme MB.

### Secondary End Points

The isolated components of the primary composite end points did not show significant differences with 1.2% and 0.7% all-cause mortality rate (OR, 0.6; 95% CI, 0.17; 1.95; *P* = .36), 1.4% and 1.6% stroke rate (OR 1.1; 95% CI, 0.43; 2.94; *P* = .81), and 4.7% and 4.9% rethoracotomy rate (OR 1.0; 95% CI, 0.60; 1.79; *P* = .89). However, operation time was notably longer in the AoSD cohort with 220.0 ± 50.8 minutes and. 204.6 ± 53.8 minutes (95% CI, 9.41; 21.38; *P* < .01). Biochemical parameters and cardiac enzymes did not present any substantial differences (creatinine-kinase_max_ [*P* = .15], creatinine-kinase isoenzyme MB_max_ [*P* = .61], Troponin_max_ [*P* = .61], Creatinine_max_ [*P* = .38]). All results in the matched and unmatched cohorts are summarized in Table 2.

### Center-Based Subgroups

In both centers, similar results to the total cohort were observed with no significant differences in the primary composite end point (center-1 [*P* = .67] and center-2 [*P* = .98]). Operation time differed in both centers with longer operation times in the AoSD cohorts, while the difference was more pronounced in center-2 (Table 3, unmatched results in Table E2).

**Table 3:** Center-based subgroup analysis of the AoSD and AAP groups

Variable	Center 1				Center 2			
	AoSD, n (%) 246 (42.8)	AAP, n (%) 228 (39.7)	OR (95% CI)	*P* value	AoSD, n (%) 329 (57.2)	AAP, n (%) 347 (60.3)	OR (95% CI)	*P* value
Composite end point	14 (5.7)	11 (4.8)	0.8 (0.37-1.89)	.67	22 (6.7)	23 (6.6)	1.0 (0.54-1.81)	.98
All-cause mortality	3 (1.2)	2 (0.9)	0.7 (0.12-4.33)	.72	4 (1.2)	2 (0.6)	0.5 (0.09-2.59)	.38
Stroke	4 (1.6)	4 (1.8)	1.1 (0.27-4.37)	.91	4 (1.2)	5 (1.4)	1.2 (0.32-4.46)	.80
Rethoracotomy	11 (4.5)	8 (3.5)	0.8 (0.31-1.97)	.59	16 (4.9)	20 (5.8)	1.2 (0.61-2.35)	.60
Operation time, min	221.6 ± 52.4	211.8 ± 58.7	−0.25 to 19.85	.06	218.8 ± 49.6	200.0 ± 50.0	11.27-26.31	**<.01**
Dialysis	4 (1.6)	5 (2.2)	1.4 (0.36-5.12)	.65	13 (4.0)	16 (4.6)	1.2 (0.55-2.47)	.68
Need for ICA	5 (2.0)	6 (2.6)	1.3 (0.39-4.33)	.67	16 (4.9)	14 (4.0)	0.8 (0.39-1.71)	.60
CPR	6 (2.4)	5 (2.2)	0.9 (0.27-2.98)	.86	9 (2.7)	7 (2.0)	0.7 (0.27-1.99)	.54
IABP	2 (0.8)	3 (1.3)	1.6 (0.27-9.82)	.59	1 (0.3)	2 (0.6)	1.9 (0.17-21.07)	.59
ECLS	4 (1.6)	2 (0.9)	0.5 (0.10-2.95)	.47	1 (0.3)	0.0 (0.0)	0.5 (0.45-0.53)	.30
PE	11 (4.5)	9 (4.0)	0.9 (0.36-2.17)	.79	7 (2.1)	10 (2.9)	1.4 (0.51-3.63)	.53
ICU stay, d	2.5 ± 6.7	2.4 ± 5.9	−1.07 to 1.21	.90	2.3 ± 4.8	2.7 ± 5.3	−1.21 to 0.32	.26
Enzymes								
CK_max_, U/L	850.1 ± 1087.9	809.7 ± 717.5	−127.33 to 208.1	.64	712.7 ± 784.9	876.4 ± 1055.6	−305.42 to 22.13	**.02**
CK-MB_max_, U/L	43.6 ± 55.6	40.7 ± 60.6	−7.64 to 13.36	.59	23.2 ± 46.3	24.5 ± 55.7	−10.23 to 7.62	.78
Troponin_max_, pg/mL	48.2 ± 108.5	54.3 ± 105.2	−25.36 to 13.26	.54	5870.1 ± 19,488.9	6371.2 ± 21,550.0	−3609.5 to 2607.23	.75
Creatinine_max_, mg/dL	0.9 ± 0.4	0.9 ± 0.4	−0.09 to 0.05	.68	1.4 ± 1.2	1.5 ± 1.0	−0.21 to 0.12	.62

*P* values < .05 presented in bold.

Composite end point = all-cause mortality, stroke, rethoracotomy. *AoSD*, Aortic sealing device; *AAP*, automated anastomosis punching; *OR*, odds ratio; *95% CI*, 95% confidence interval; *ICA*, invasive coronary angiography; *CPR*, cardiopulmonary resuscitation; *IABP*, intra-aortic balloon pump; *ECLS*, extracorporeal life support; *PE*, pericardial effusion; *ICU*, intensive care unit; *CK*, creatinine kinase; *CK-MB*, creatinine kinase isoenzyme MB.

### Cardiac Function–Based Subgroups

In both subgroups, similar results to the total cohort were observed with no significant differences in the primary composite end point (ejection fraction ≤45% [*P* = .58] and ejection fraction >45% [*P* = .91]). The variation in operation time within the total cohort, was observed in patients with preserved ejection fraction (*P* < .01); however, the operation time in patients with an impaired ejection fraction was longer regardless of the used CAA device than in patients with preserved contractility (Table 4, unmatched results in Table E3).

**Table 4:** Contractility-based subgroup analysis of the AoSD and AAP groups

Variable	EF <45%				EF ≥45%			
	AoSD, n (%) 65 (11.3)	AAP, n (%) 102 (17.7)	OR (95% CI)	*P* value	AoSD, n (%) 510 (98.7)	AAP, n (%) 473 (82.3)	OR (95% CI)	*P* value
Composite end point	6 (9.2)	7 (6.9)	0.7 (0.23-2.26)	.58	30 (5.9)	27 (5.7)	1.0 (0.57-1.65)	.91
All-cause mortality	2 (3.1)	2 (2.0)	0.6 (0.09-4.59)	.65	5 (1.0)	2 (0.4)	0.4 (0.08-2.22)	.30
Stroke	2 (3.1)	1 (1.0)	0.3 (0.03-3.51)	.32	6 (1.2)	8 (1.7)	1.5 (0.50-4.20)	.50
Re-thoracotomy	5 (7.7)	6 (5.9)	0.8 (0.22-2.57)	.65	22 (4.3)	22 (4.7)	1.1 (0.59-1.98)	.80
Operation time (min)	215.3 ± 50.7	208.4 ± 55.7	−10.0 to 23.80	.42	220.6 ± 50.8	203.8 ± 53.5	10.21-23.27	**<.01**
Dialysis	3 (4.6)	9 (8.8)	2.0 (0.52-7.68)	.31	14 (2.8)	12 (2.5)	0.9 (0.42-2.01)	.84
Need for ICA	2 (3.1)	2 (2.0)	0.6 (0.09-4.59)	.65	19 (3.7)	18 (3.8)	1.0 (0.53-1.97)	.95
CPR	1 (1.5)	4 (3.9)	2.6 (0.29-23.90)	.38	14 (2.8)	8 (1.7)	0.6 (0.25-1.47)	.26
IABP	0 (0.0)	3 (2.9)	0.6 (0.53-0.68)	.16	3 (0.6)	2 (0.4)	0.7 (0.12-4.31)	.72
ECLS	2 (3.1)	1 (1.0)	0.3 (0.03-3.51)	.32	3 (0.6)	1 (0.2)	0.4 (0.04-3.45)	.35
PE	2 (3.1)	8 (7.8)	2.7 (0.55-13.04)	.21	16 (3.1)	11 (2.3)	0.7 (0.34-1.60)	.44
ICU stay, d	3.6 ± 7.8	4.0 ± 8.6	−3.07 to 2.12	.72	2.3 ± 5.3	2.3 ± 4.6	−0.71 to 0.54	.81
Enzymes								
CK_max_, U/L	831.0 ± 1053.6	894.7 ± 1095.0	−402.53 to 275.06	.71	763.9 ± 912.0	839.8 ± 896.8	−189.73 to 37.83	.19
CK-MB_max_, U/L	32.5 ± 64.9	30.0 ± 62.8	−19 to 23.94	.82	32.5 ± 49.8	33.5 ± 58.0	−8.38 to 6.24	.77
Troponin_max_, pg/mL	5116.5 ± 25,768.1	5876.1 ± 26,004.0	−8879.72 to 7360.45	.85	3157.9 ± 13,046.4	3433.0 ± 14,363.5	−1990.78 to 1440.59	.75
Creatinine_max_, mg/dL	1.1 ± 0.4	1.6 ± 1.3	−0.88 to −0.23	**<.01**	1.2 ± 1.0	1.2 ± 0.7	−0.07 to 0.15	.38

*P* values < .05 presented in bold.

Composite end point = all-cause mortality, stroke, rethoracotomy. *EF*, Ejection fraction; *AoSD*, aortic sealing device; *AAP*, automated anastomosis punching; *OR*, odds ratio; *95% CI*, 95% confidence interval; *ICA*, invasive coronary angiography; *CPR*, cardiopulmonary resuscitation; *IABP*, intra-aortic balloon pump; *ECLS*, extracorporeal life support; *PE*, pericardial effusion; *ICU*, intensive care unit; *CK*, creatinine kinase; *CK-MB*, creatinine kinase isoenzyme MB.

### Age-Based Subgroups

In both age groups (>70 years and ≤70 years), similar results to the total cohort were observed with no significant differences in the primary composite end point (>70 years [*P* = .95] and ≤70 years [*P* = . 60]) or the perioperative outcome and enzymes. The operation time was longer in both age groups in the AoSD group (>70 years [*P* < .01] and ≤70 years [*P* < .01]) (Table 5, unmatched results in Table E4).

**Table 5:** Age-based subgroup analysis of the AoSD and AAP groups

Variable	Age >70 years				Age ≤70 years			
	AoSD, n (%) 265 (46.1)	AAP, n (%) 272 (47.3)	OR (95% CI)	*P* value	AoSD, n (%) 310 (53.9)	AAP, n (%) 303 (52.7)	OR (95% CI)	*P* value
Composite end point	21 (7.9)	17 (6.3)	0.8 (0.40-1.50)	.45	15 (4.8)	17 (5.6)	1.2 (0.57-2.39)	.67
All-cause mortality	6 (2.3)	4 (1.5)	0.6 (0.18-2.31)	.50	1 (0.3)	0 (0.0)		.32
Stroke	5 (1.9)	8 (2.9)	1.6 (0.51-4.88)	.43	3 (1.0)	1 (0.3)	0.3 (0.04-3.28)	.33
Rethoracotomy	13 (4.9)	12 (4.4)	0.9 (0.40-2.00)	.79	14 (4.5)	16 (5.3)	1.2 (0.56-2.46)	.66
Operation time, min	208.6 ± 46.9	194.7 ± 54.2	5.24-22.46	**<.01**	229.7 ± 51.9	213.4 ± 52.1	8.00-24.51	**<.01**
Dialysis	9 (3.4)	12 (4.4)	1.3 (0.54-3.16)	.55	8 (2.6)	9 (3.0)	1.2 (0.44-3.04)	.77
Need for ICA	8 (3.0)	9 (3.3)	1.1 (0.42-2.89)	.85	13 (4.2)	11 (3.6)	0.9 (0.38-1.95)	.72
CPR	10 (3.8)	8 (2.9)	0.8 (0.30-1.99)	.59	5 (1.6)	4 (1.3)	0.8 (0.22-3.07)	.76
IABP	3 (1.1)	4 (1.5)	1.3 (0.29-5.88)	.73	0 (0.0)	1 (0.3)		.31
ECLS	2 (0.8)	2 (0.7)	1.0 (0.14-6.97)	.98	3 (1.0)	0 (0.0)		.09
PE	8 (3.0)	9 (3.3)	1.1 (0.42-2.90)	.84	10 (3.2)	10 (3.3)	1.0 (0.42-2.50)	.96
ICU stay, d	3.0 ± 6.4	3.1 ± 6.4	−1.21 to 0.96	.82	1.9 ± 4.9	2.2 ± 4.6	−1.07 to 0.45	.43
Enzymes								
CK_max_, U/L	685.6 ± 720.9	801.9 ± 869.0	−252.00 to 19.48	.09	844.8 ± 1070.2	893.0 ± 989.4	−212.63 to 116.33	.57
CK-MB_max_, U/L	31.9 ± 45.6	39.2 ± 74.7	−18.42 to 3.93	.20	33.0 ± 56.5	27.3 ± 38.4	−2.90 to 14.2	.18
Troponin_max_, pg/mL	2924.9 ± 14,903.8	4263.9 ± 17,853.6	−4130.60 to 1452.58	.35	3767.8 ± 15,116.7	3509.6 ± 16,246.6	−2230.25 to 2746.69	.84
Creatinine_max_, mg/dL	1.3 ± 1.1	1.3 ± 0.7	−0.17 to 0.15	.94	1.1 ± 0.8	1.2 ± 0.9	−0.22 to 0.06	.27

*P* values < .05 presented in bold.

Composite end point = all-cause mortality, stroke, rethoracotomy. *AoSD*, Aortic sealing device; *AAP*, automated anastomosis punching; *OR*, odds ratio; *95% CI*, 95% confidence interval; *ICA*, invasive coronary angiography; *CPR*, cardiopulmonary resuscitation; *IABP*, intra-aortic balloon pump; *ECLS*, extracorporeal life support; *PE*, pericardial effusion; *ICU*, intensive care unit; *CK*, creatinine kinase; *CK-MB*, creatinine kinase isoenzyme MB.

## Discussion

**Figure 3:**
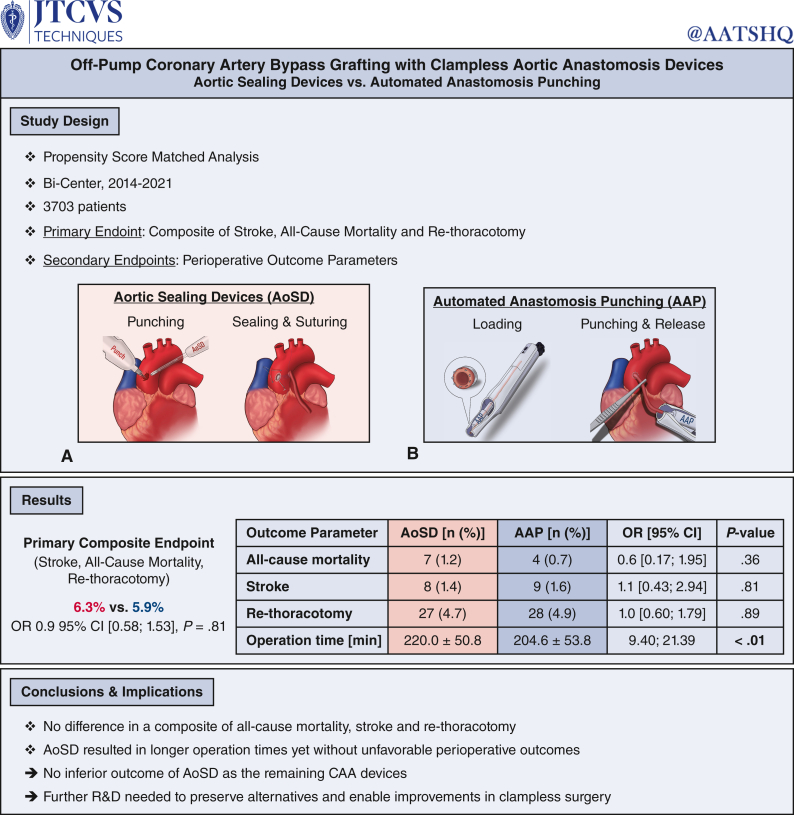
Off-pump coronary artery bypass grafting with clampless aortic anastomosis devices aortic sealing devices versus automated anastomosis punching. *AoSD*, Aortic sealing device; *AAP*, automated anastomosis punching; *OR*, Odds ratio; *95% CI*, 95% confidence intervals; *CAA*, clampless aortic device; *R&D*, research and development.

This is the first study to perform a large-scale analysis of the perioperative outcomes of both major representatives of clampless aortic anastomosis devices. The results are 3-fold: (1) there is no difference regarding a composite of all-cause mortality, stroke, and rethoracotomy; (2) nor in the perioperative outcomes; and (3) although the use of AoSD resulted in longer operation times, it did not correlate with an unfavorable perioperative outcome (Figure 3).

Our data underline the effectiveness and safety of both systems with regard to perioperative outcome data implying that, by the only remaining approach of AoSD systems, there is no hint of inadequate care for patients undergoing clampless OPCAB surgery.

Neurologic complications are by far the most assessed complication and therefore main advantage in association with clampless surgery^4^^,^^6^^,^^20^ since aortic manipulation remains an independent risk factor for postoperative stroke^23^^,^^24^ despite its multifactorial origin.^25^ However, we decided to primarily evaluate a combined end point of all-cause mortality, stroke, and rethoracotomy, which allows us to assess the main effect (reduction in stroke) and complications (bleeding, mortality) of the compared systems overall. In addition, we believe that in the presented analysis the in-hospital “short-term” period is of the greatest interest since the benefits or flaws of the systems are most likely to occur in this period.

One major difference between both systems is the need to perform manual anastomosis, which resulted in a longer operation time in AoSD (220.0 ± 50.8 minutes vs 204.6 ± 53.8 minutes) and is in concordance with further data assessing AAP systems.^17^ In contrast, since the anastomosis gained by the AAP device could not be cobra-head-like shaped and angled, but had to be perpendicular to the ascending aorta, it was under suspicion to have a less-qualified central anastomosis, which could be disproved.^21^^,^^26^

The results regarding OPCAB surgery in general have to be interpreted carefully, since the recent 5-year results of the GOPCABE (German Off-Pump Coronary Artery Bypass Grafting in Elderly Patients) trial did show no difference between conventional coronary artery bypass grafting (cCABG) and OPCAB.^27^ These results are in line with the CORONARY (Coronary Artery Bypass Surgery (CABG) Off or On Pump Revascularization Study) and ROOBY (Randomized On/Off Cardiopulmonary Bypass) trials,^28^^,^^29^ where the OPCAB cohorts consisted of a mix of no-aortic-touch, CAA-device-assisted clampless, and partial clamped cases. With all, there was no in-depth analysis of clampless OPCAB approaches, which was discussed by Reents and colleagues^30^ for GOPCABE. Comparisons between cCABG and OPCAB have yielded diverse findings in various works presenting “benefits”^5^^,^^6^^,^^10^^,^^24^ and “no benefits”^27^^,^^31^^,^^32^ of the OPCAB procedure, prohibiting a clear recommendation in favor of or against the technique.^1^ However, the best results could be presented when OPCAB was performed with a clampless approach.^5^ Thus, the often-mentioned benefit of OPCAB, to avoid ECC, seems to be of less clinical impact. Although our data with regard to stroke (AoSD [1.4%], AAP [1.6%]) and mortality (AoSD [1.2%], AAP [0.7%]) did not differ statistically, the results are in line with the nationwide report for cardiac surgery in Germany 2021 considering in-hospital stroke (1.7%) and mortality (3.3%).^33^ Recently, the 10-year results of the ART trial presented no superiority of a multiarterial approach and no differences between OPCAB and cCABG in subgroup analyses.^34^ The results of the ROMA (Randomized comparison of the clinical Outcome of single versus Multiple Arterial grafts) trial (NCT03217006), an ongoing large-scale study regarding CABG surgery with single arterial grafts and multiple arterial grafts, are still pending. Regrettably, both major trials did not assess the application of particular clampless devices, which is elucidated within the framework of our study.

Although multiple studies have shown the efficacy of CAA devices, yielding outcomes comparable with no-aortic-touch techniques,^5^^,^^21^ additional research and development in this field are crucial. Nonetheless, advancements within a restricted scope have been limited. With only 4 primary systems employed over the past 2 decades, and currently, only 2 of these systems persist, both following the same AoSD approach, which still exhibits the capability to dislodge atheromatous material.^20^ The guidelines recommendations of diagnostic tools for risk adjustment such as epiaortic ultrasound,^1^ which could prove superior to digital palpation,^35^ might surely be reasonable but lack in ubiquitous availability.^18^ Notwithstanding its effectiveness, the systemic scan of the aorta might help only to dodge aortic regions of danger such as calcifications but does not replace future devices that may provide further solutions for stroke and major adverse events prevention.

Thus, in conclusion the implications of the presented data have to be 2-fold. In light of the absence of disparity regarding the primary end point, the current limitation to AoSD exclusivity is not translating to a detriment to overall outcomes. However, the data present solid results for AAP systems and no kind of inferiority, which should encourage research and development on either ways or even new approaches that are yet to be discovered, to improve and draw the future of clampless bypass surgery.

### Limitations

The primary limitation of the analysis resides in its retrospective study design, emphasizing the imperative for validation by prospective multicenter-controlled randomized trials. The AAP group depended on only one system, whereas for AoSD only the Heartstring III System (Getinge Group) was used, with limited transferability of the presented data to other AoSD devices like the Enclose II System (Peters Surgical). A *P*-value adjustment in subgroup analysis was not performed due to small sample sizes and a high probability of type 1 error, yet, a type 2 error cannot be ruled out. Regarding safety analysis, the described bail-out strategy to perform partial clamping, if device-related complications occurred, was not part of the analysis due to missing data. The outcome “need for invasive coronary angiography (ICA)” is not dependent on any adverse diagnosis of the ICA but the perioperative outcome of a clinical patient constitution leading to the decision to perform ICA. Hence, the analysis did not compass perioperative anastomosis failure.

## Conclusions

The CAA strategies AoSD and AAP did not differ in perioperative outcome parameters, whereas the implementation of AoSD was associated with a prolonged operation time without inducing any inferior clinical outcome.

## Conflict of Interest Statement

The authors reported no conflicts of interest.

The *Journal* policy requires editors and reviewers to disclose conflicts of interest and to decline handling or reviewing manuscripts for which they may have a conflict of interest. The editors and reviewers of this article have no conflicts of interest.

## Figures and Tables

**Table E1:** Logistic regression of the primary end point in the AoSD and AAP groups in the unmatched cohorts

Variable	Unmatched cohort				
	AoSD, n (%) 265 (46.1)	AAP, n (%) 1584 (50.6)	OR (95% CI)	*P* value	Wald
Composite end point, n (%)	36 (6.3)	143 (4.6)	0.6 (0.18-1.94)	.38	0.76
All-cause mortality, n (%)	7 (1.2)	18 (0.6)	0.7 (0.22-2.09)	.51	0.45
Stroke, n (%)	8 (1.4)	32 (1.0)	1.1 (0.39-3.30)	.82	0.06
Rethoracotomy, n (%)	27 (4.7)	116 (3.7)	1.3 (0.43-4.19)	.61	0.26

Composite end point = all-cause mortality, stroke, rethoracotomy. *AoSD*, Aortic sealing device; *AAP*, automated anastomosis punching; *OR*, odds ratio; *95% CI*, 95% confidence interval.

**Table E2:** Center-based subgroup analysis of the AoSD and AAP group in the unmatched cohorts

Variable	Center 1				Center 2			
	AoSD, n (%) 246 (42.8)	AAP, n (%) 239 (7.6)	OR (95% CI)	*P* value	AoSD, n (%) 329 (57.2)	AAP, n (%) 2889 (92.4)	OR (95% CI)	*P* value
Composite end point, n (%)	14 (5.7)	11 (4.6)	0.8 (0.36-1.80)	.59	22 (6.7)	132 (4.6)	0.7 (0.42-1.07)	.09
All-cause mortality, n (%)	3 (1.2)	2 (0.8)	0.7 (0.11-4.13)	.68	4 (1.2)	16 (0.6)	0.5 (0.15-1.36)	.15
Stroke, n (%)	4 (1.6)	4 (1.7)	1.0 (0.25-4.17)	.97	4 (1.2)	28 (1.0)	0.8 (0.28-2.28)	.67
Rethoracotomy, n (%)	11 (4.5)	8 (3.4)	0.7 (0.29-1.87)	.52	16 (4.9)	108 (3.7)	0.8 (0.44-1.30)	.32
Operation time, min	221.6 ± 52.4	212.8 ± 58.2	−1.07 to 18.72	.08	218.8 ± 49.6	199.2 ± 46.3	14.23-24.88	**<.01**
Dialysis, n (%)	4 (1.6)	5 (2.1)	1.3 (0.34-4.87)	.70	13 (4.0)	118 (4.1)	1.0 (0.58-1.85)	.92
Need for ICA, n (%)	5 (2.0)	6 (2.5)	1.2 (0.37-4.12)	.72	16 (4.9)	89 (3.1)	0.6 (0.36-1.07)	.08
CPR, n (%)	6 (2.4)	5 (2.1)	0.9 (0.26-2.84)	.80	9 (2.7)	49 (1.7)	0.6 (0.03-1.26)	.18
IABP, n (%)	2 (0.8)	3 (1.3)	1.6 (0.26-9.36)	.63	1 (0.3)	10 (0.4)	1.1 (0.15-8.93)	.90
ECLS, n (%)	4 (1.6)	2 (0.8)	0.5 (0.09-2.81)	.43	1 (0.3)	12 (0.4)	1.4 (0.18-10.56)	.76
PE, n (%)	11 (4.5)	10 (4.2)	0.9 (0.39-2.25)	.88	7 (2.1)	53 (1.8)	0.9 (0.39-1.91)	.71
ICU stay, d	2.5 ± 6.7	2.4 ± 5.8	−1.02 to 1.21	.86	2.3 ± 4.8	2.4 ± 5.0	−0.66 to 0.48	.78
Enzymes								
CK_max_, U/L	850.1 ± 1087.9	805.3 ± 712.6	−119.81 to 209.38	.59	712.7 ± 784.9	828.6 ± 1040.5	−232.17 to 0.19	.02
CK-MB_max_, U/L	43.6 ± 55.6	40.6 ± 59.3	−7.26 to 13.28	.56	23.2 ± 46.3	25.5 ± 61.2	−9.60 to 5.06	.55
Troponin_max_, pg/mL	48.2 ± 108.5	54.6 ± 104.4	−25.39 to 12.62	.51	5870.1 ± 19,488.9	6102.0 ± 25,220.1	−3049.43 to 2585.71	.87
Creatinine_max_, mg/dL	0.9 ± 0.4	0.9 ± 0.4	−0.09 to 0.05	.66	1.4 ± 1.2	1.5 ± 1.1	−0.21 to 0.05	.25

*P* values < .05 presented in bold.

Composite end point = all-cause mortality, stroke, rethoracotomy. *AoSD*, Aortic sealing device; *AAP*, automated anastomosis punching; *OR*, odds ratio; *95% CI*, 95% confidence interval; *ICA*, invasive coronary angiography; *CPR*, cardiopulmonary resuscitation; *IABP*, intra-aortic balloon pump; *ECLS*, extracorporeal life support; *PE*, pericardial effusion; *ICU*, intensive care unit; *CK*, creatinine kinase; *CK-MB*, creatinine kinase isoenzyme MB.

**Table E3:** Contractility-based subgroup analysis of the AoSD and AAP group in the unmatched cohorts

Variable	EF <45%				EF ≥45%			
	AoSD, n (%) 65 (11.3)	AAP, n (%) 406 (13.0)	OR (95% CI)	*P* value	AoSD, n (%) 510 (98.7)	AAP, n (%) 2722 (87.0)	OR (95% CI)	*P* value
Composite end point, n (%)	6 (9.2)	20 (4.9)	0.5 (0.20-1.32)	.16	30 (5.9)	123 (4.5)	0.8 (0.50-1.14)	.18
All-cause mortality, n (%)	2 (3.1)	5 (1.2)	0.4 (0.07-2.07)	.25	5 (1.0)	13 (0.5)	0.5 (0.17-1.37)	.16
Stroke, n (%)	2 (3.1)	5 (1.2)	0.4 (0.07-2.07)	.25	6 (1.2)	27 (1.0)	0.8 (0.35-2.05)	.70
Rethoracotomy, n (%)	5 (7.7)	16 (3.9)	0.5 (0.17-1.39)	.17	22 (4.3)	100 (3.7)	0.9 (0.53-1.36)	.49
Operation time, min	215.3 ± 50.7	200.5 ± 47.3	2.29-27.37	.02	220.6 ± 50.8	200.2 ± 47.5	15.83-24.92	**<.01**
Dialysis, n (%)	3 (4.6)	31 (7.6)	1.7 (0.51-5.76)	.38	14 (2.8)	92 (3.4)	1.2 (0.70-2.19)	.46
Need for ICA, n (%)	2 (3.1)	16 (3.9)	1.3 (0.29-5.76)	.74	19 (3.7)	79 (2.9)	0.8 (0.46-1.29)	.32
CPR, n (%)	1 (1.5)	11 (2.7)	1.8 (0.23-14.04)	.58	14 (2.8)	43 (1.6)	0.6 (0.31-1.05)	.07
IABP, n (%)	0 (0.0)	6 (1.5)	0.9 (0.83-0.89)	.32	3 (0.6)	7 (0.3)	0.4 (0.11-1.69)	.22
ECLS, n (%)	2 (3.1)	4 (1.0)	0.3 (0.06-1.75)	.16	3 (0.6)	10 (0.4)	0.6 (0.17-2.27)	.47
PE, n (%)	2 (3.1)	11 (2.7)	0.9 (0.19-4.05)	.87	16 (3.1)	52 (1.9)	0.6 (0.34-1.06)	.08
ICU stay, d	3.6 ± 7.8	3.5 ± 7.1	−1.82 to 1.94	.95	2.3 ± 5.3	2.2 ± 4.7	−0.43 to 0.48	.91
Enzymes								
CK_max_, U/L	831.0 ± 1053.6	891.0 ± 1393.6	−415.48 to 295.46	.74	763.9 ± 912.0	817.2 ± 949.8	−142.86 to 36.15	.24
CK-MB_max_, U/L	32.5 ± 64.9	26.5 ± 54.8	−9.78 to 21.76	.45	32.5 ± 49.8	27.5 ± 62.1	−1.14 to 110.00	.11
Troponin_max_, pg/mL	5116.5 ± 25,768.1	5312.2 ± 15,115.3	−4650.10 to 4258.61	.93	3157.9 ± 13,046.4	5688.6 ± 25,377.9	−4788.13 to −273.23	**.03**
Creatinine_max_, mg/dL	1.1 ± 0.4	1.8 ± 1.5	−1.05 to −0.34	**<.01**	1.2 ± 1.0	1.4 ± 1.0	−0.28 to −0.09	**<.01**

*P* values < .05 presented in bold.

Composite end point = all-cause mortality, stroke, rethoracotomy. *EF*, Ejection fraction; *AoSD*, aortic sealing device; *AAP*, automated anastomosis punching; *OR*, odds ratio; *95% CI*, 95% confidence interval; *ICA*, invasive coronary angiography; *CPR*, cardiopulmonary resuscitation; *IABP*, intra-aortic balloon pump; *ECLS*, extracorporeal life support; *PE*, pericardial effusion; *ICU*, intensive care unit; *CK*, creatinine kinase; *CK-MB*, creatinine kinase isoenzyme MB.

**Table E4:** Age-based subgroup analysis of the AoSD and AAP groups in the unmatched cohorts

Variable	Age >70 years				Age ≤70 years			
	AoSD, n (%) 265 (46.1)	AAP, n (%) 1584 (50.6)	OR (95% CI)	*P* value	AoSD, n (%) 310 (53.9)	AAP, n (%) 1541 (49.4)	OR (95% CI)	*P* value
Composite end point, n (%)	21 (7.9)	77 (4.9)	0.6 (0.36-0.98)	**.039**	15 (4.8)	66 (4.3)	0.9 (0.50-1.56)	.66
All-cause mortality, n (%)	6 (2.3)	12 (0.8)	0.3 (0.12-0.88)	**.021**	1 (0.3)	6 (0.4)	1.2 (0.14-10.07)	.86
Stroke, n (%)	5 (1.9)	23 (1.5)	0.8 (0.29-2.03)	.59	3 (1.0)	9 (0.6)	0.6 (0.16-2.23)	.44
Rethoracotomy, n (%)	13 (4.9)	59 (3.7)	0.8 (0.40-1.38)	.35	14 (4.5)	57 (3.7)	0.8 (0.45-1.48)	.49
Operation time, min	208.6 ± 46.9	191.1 ± 44.7	11.63-23.36	**<.01**	229.7 ± 51.9	209.6 ± 48.3	14.09-26.05	**<.01**
Dialysis, n (%)	9 (3.4)	76 (4.8)	1.4 (0.71-2.88)	.32	8 (2.6)	47 (3.1)	1.2 (0.56-2.54)	.66
Need for ICA, n (%)	8 (3.0)	50 (3.2)	1.1 (0.49-2.23)	.91	13 (4.2)	45 (2.9)	0.7 (0.37-1.29)	.24
CPR, n (%)	10 (3.8)	30 (1.9)	0.5 (0.24-1.02)	.05	5 (1.6)	24 (1.6)	1.0 (0.37-2.55)	.94
IABP, n (%)	3 (1.1)	9 (0.6)	0.5 (0.13-1.85)	.29	0 (0.0)	4 (0.3)		.37
ECLS, n (%)	2 (0.8)	8 (0.5)	0.7 (0.14-3.15)	.61	3 (1.0)	6 (0.4)	0.4 (0.10-1.61)	.18
PE, n (%)	8 (3.0)	36 (2.3)	0.8 (0.34-1.62)	.46	10 (3.2)	27 (1.8)	0.5 (0.26-1.11)	.09
ICU stay, d	3.0 ± 6.4	2.7 ± 6.0	−0.58 to 1.00	.59	1.9 ± 4.9	2.0 ± 3.8	−0.60 to 0.38	.67
Enzymes								
CK_max_, U/L	685.6 ± 720.9	756.4 ± 1023.7	−199.27 to 57.77	.28	844.8 ± 1070.2	899.0 ± 1008.2	−178.84 to 70.56	.39
CK-MB_max_, U/L	31.9 ± 45.6	29.3 ± 68.6	−6.42 to 11.56	.57	33.0 ± 56.5	25.4 ± 52.3	0.54 to 14.60	**.03**
Troponin_max_, pg/mL	2924.9 ± 14,903.8	5715.5 ± 22,136.4	−5557.01 to −24.14	**.05**	3767.8 ± 15,116.7	5561.8 ± 26,330.4	−4823.07 to 1235.07	.25
Creatinine_max_, mg/dL	1.3 ± 1.1	1.5 ± 1.0	−0.37 to −0.11	**<.01**	1.1 ± 0.8	1.3 ± 1.2	−0.38 to −0.11	**<.01**

*P* values < .05 presented in bold.

Composite end point = all-cause mortality, stroke, rethoracotomy. *AoSD*, Aortic sealing device; *AAP*, automated anastomosis punching; *OR*, odds ratio; *95% CI*, 95% confidence interval; *ICA*, invasive coronary angiography; *CPR*, cardiopulmonary resuscitation; *IABP*, intra-aortic balloon pump; *ECLS*, extracorporeal life support; *PE*, pericardial effusion; *ICU*, intensive care unit; *CK*, creatinine kinase; *CK-MB*, creatinine kinase isoenzyme MB.
